# *In Utero* Exposure to Diesel Exhaust Air Pollution Promotes Adverse Intrauterine Conditions, Resulting in Weight Gain, Altered Blood Pressure, and Increased Susceptibility to Heart Failure in Adult Mice

**DOI:** 10.1371/journal.pone.0088582

**Published:** 2014-02-12

**Authors:** Chad S. Weldy, Yonggang Liu, H. Denny Liggitt, Michael T. Chin

**Affiliations:** 1 Division of Cardiology, Department of Medicine, University of Washington School of Medicine, Seattle, Washington, United States of America; 2 Department of Pathology, University of Washington School of Medicine, Seattle, Washington, United States of America; 3 Department of Comparative Medicine, University of Washington School of Medicine, Seattle, Washington, United States of America; Virginia Commonwealth University, United States of America

## Abstract

Exposure to fine particulate air pollution (PM_2.5_) is strongly associated with cardiovascular morbidity and mortality. Exposure to PM_2.5_ during pregnancy promotes reduced birthweight, and the associated adverse intrauterine conditions may also promote adult risk of cardiovascular disease. Here, we investigated the potential for *in utero* exposure to diesel exhaust (DE) air pollution, a major source of urban PM_2.5_, to promote adverse intrauterine conditions and influence adult susceptibility to disease. We exposed pregnant female C57Bl/6J mice to DE (≈300 µg/m^3^ PM_2.5_, 6 hrs/day, 5 days/week) from embryonic day (E) 0.5 to 17.5. At E17.5 embryos were collected for gravimetric analysis and assessed for evidence of resorption. Placental tissues underwent pathological examination to assess the extent of injury, inflammatory cell infiltration, and oxidative stress. In addition, some dams that were exposed to DE were allowed to give birth to pups and raise offspring in filtered air (FA) conditions. At 10-weeks of age, body weight and blood pressure were measured. At 12-weeks of age, cardiac function was assessed by echocardiography. Susceptibility to pressure overload-induced heart failure was then determined after transverse aortic constriction surgery. We found that *in utero* exposure to DE increases embryo resorption, and promotes placental hemorrhage, focal necrosis, compaction of labyrinth vascular spaces, inflammatory cell infiltration and oxidative stress. In addition, we observed that *in utero* DE exposure increased body weight, but counterintuitively reduced blood pressure without any changes in baseline cardiac function in adult male mice. Importantly, we observed these mice to have increased susceptibility to pressure-overload induced heart failure, suggesting this *in utero* exposure to DE ‘reprograms’ the heart to a heightened susceptibility to failure. These observations provide important data to suggest that developmental exposure to air pollution may strongly influence adult susceptibility to cardiovascular disease.

## Introduction

The inhalation of fine ambient particulate matter (PM_2.5_; PM <2.5 µm in diameter) has long been associated with increased risk of cardiovascular morbidity and mortality [1], [2], [3]. Ambient PM_2.5_ air pollution was recently listed as the ninth-ranked cause of disease worldwide, and the fourth-ranked cause of disease in East Asia [4]. To understand the pathological mechanisms that may explain these population-wide observations, investigators utilizing controlled exposure facilities and animal models have begun to elucidate potential biological mechanisms by which PM_2.5_ adversely affects systemic cardiovascular function. Studies investigating the acute as well as the chronic effects of PM_2.5_ inhalation have demonstrated that PM_2.5_ inhalation is capable of promoting multiple deleterious effects on the cardiovascular system through a variety of mechanisms, including: 1) inciting pulmonary inflammation, resulting in the spillover of reactive products into the circulation, including proinflammatory cytokines, 2) dysregulating the autonomic nervous system, and 3) directly translocating particles from the lung into the circulation, influencing vascular inflammation and function [5]. All of these pathways have been suggested to play a role in the observed acceleration of atherosclerosis [6], [7], [8], [9], impaired vascular function and loss of bioavailable nitric oxide (NO) [10], [11], and induction of cardiac remodeling and increased susceptibility to heart failure [12], [13] in rodent PM_2.5_ exposure models.

As the inhalation of PM_2.5_ can have adverse effects on systemic vascular function, there has been an increased interest to understand the potential effect of *in utero* PM_2.5_ exposure on the developing fetus. During development, the highly vascularized placenta is the primary component of the fetomaternal interface, regulating oxygen tension, nutrient levels, certain immune functions, and intrauterine growth [14], [15], [16]. In a recent multicenter meta-analysis investigating the effect of PM_10_ and PM_2.5_ exposure during pregnancy and birthweight, Dadvand and colleagues (2013) reported PM exposure to be associated with reduced birthweight, as both a term low-birthweight outcome variable and as a continuous variable [17]. These data provide evidence that PM exposure can adversely affect intrauterine growth, likely in a linear manner. As much of traffic related PM_2.5_ is derived from diesel exhaust (DE) [18], controlled DE exposures using mouse models have demonstrated *in utero* DE exposure to promote placental and fetal inflammation in late gestation [19], [20]. These effects of DE on *in utero* inflammation are associated with adult susceptibility to weight gain and obesity [20] as well as airway hyperreactivity following subsequent ozone exposure [19].

Barker and colleagues have previously revealed fetal development to be a critical window that strongly influences adult susceptibility to disease, leading to the concept of ‘fetal origins of adult disease’ [21], [22], [23], [24], [25], [26], [27]. Importantly, reduced birthweight has been observed to be associated with increased adult risk of cardiovascular mortality [21] as well as increased ventricular hypertrophy [27] and hypertension [26], suggesting that adverse intrauterine conditions that promote reduced birthweight, may also promote lifelong susceptibility to cardiovascular diseases that increase overall risk of cardiovascular mortality. In addition, Eriksson and colleagues (2000) reported that reduced placental weight was associated with an increased risk of hypertension in patients with diabetes [22], suggesting that placental insufficiency may exert a profound influence on adult cardiovascular physiology.

In our previous work, we compared the effects of ‘adult’ versus ‘developmental’ exposure to DE on adult susceptibility to heart failure in mice [28]. Interestingly, we observed that offspring exposed to DE *in utero* and early in life, where exposure began maternally 3-weeks prior to pregnancy, throughout pregnancy, and until offspring were 3 weeks of age, had increased sensitivity to pressure overload-induced heart failure as adults. This effect was not observed in mice exposed to DE for two months post weaning, suggesting that developmental exposure to DE produces long lasting effects on adult myocardial susceptibility to injury. Although our previous work demonstrates the importance of developmental exposures to air pollution on adult susceptibility to disease, it did not determine the potential for *in utero* exposure to air pollution alone to influence adult health. Understanding the potential for *in utero* air pollution exposure to influence adult cardiovascular risk will likely have strong public health implications.

From these prior observations, we hypothesized that *in utero* exposure to DE air pollution would promote placental oxidative stress and inflammation, resulting in adverse intrauterine conditions leading to increased embryo resorption as well as decreased placental and fetal weights. We also hypothesized that *in utero* exposure would promote weight gain, increased blood pressure, and increased susceptibility to pressure overload induced heart failure in adult mice. To test these hypotheses, we exposed pregnant female C57BL6/J mice to DE (300 µg/m^3^ PM_2.5_, 6 hrs/day, 5 days/week) or filtered air (FA) from embryonic day 0.5 (E0.5) through E17.5, at which point we assessed embryo resorption, placental and fetal weights, placental oxidative stress as measured by 3-nitrotyrosine protein modification, placental histology by hematoxylin and eosin (H&E) staining, and inflammatory cell infiltration by α-CD45 immunohistochemistry. In addition, following birth, offspring were raised in FA conditions to adulthood for subsequent measurement of adult body weight, blood pressure, cardiac function and susceptibility to pressure overload-induced heart failure. In this study, we report that *in utero* DE exposure promotes an adverse intrauterine environment that results in adult weight gain, altered blood pressure and increased susceptibility to pressure overload-induced heart failure.

## Materials and Methods

### Ethics Statement

All animal work was conducted according to relevant national and international guidelines. This study was carried out in strict accordance with the recommendations in the Guide for the Care and Use of Laboratory Animals of the National Institutes of Health. All animal experiments were approved by the University of Washington Institutional Animal Care and Use Committee (PHS Animal Assurance Welfare # A3464-01).

### Diesel Exhaust Exposure and Mice

Male and female C57Bl/6J mice were purchased from The Jackson Laboratory (Bar Harbor, Maine, USA). All mice were housed in specific pathogen free (SPF) conditions on a 12/12-light/dark cycle. Female and male mice between the ages of 12 to 14 weeks were transferred to our Northlake Diesel Exposure Facility located near the University of Washington (UW) and housed under SPF conditions in Allentown caging systems (Allentown, NJ, USA) as previously described [29], [30], [31]. Diesel exhaust (DE) was generated from a single cylinder Yanmar diesel engine (Model YDG5500EV-6EI) operating on 82% load. A detailed analysis of DE particulate components in this system has been previously reported [29]. DE exposures were conducted for 6 hours per day (9 am –3 pm) five days a week (Monday – Friday) and DE concentrations were regulated to 300 µg/m^3^ of PM_2.5_. A 300 µg/m^3^ concentration of PM_2.5_ six hours/day, five days/week equates to a time weighted hourly average of 53 µg/m^3^.

Exposure characteristics detailing gas, particle-bound polycyclic aromatic hydrocarbons (PAH), and particle diameter were recently measured and reported [28], [32]. Briefly, oxides of nitrogen concentrations were 1800 ppb NO_x_ and 60 ppb NO_2_, carbon monoxide was 2 ppm, and carbon dioxide was 1000 ppm. The mass fraction of particle-bound PAH was 20 ng/µg PM_2.5_ and the ratio of the organic carbon to elemental carbon mass concentration was 0.10. The mass median aerodynamic diameter of particles was 85 nm (GSD 1.2) and the count median thermodynamic equivalent diameter was 87 nm (GSD 3.0).

### E17.5 Fetal and Placental Collection

For our assessment of the embryonic effects of *in utero* DE exposure, timed matings were initiated on Monday afternoon and subsequently checked for a visible vaginal plug the following morning. If plugs were visible, mice were immediately transferred to either FA or DE. Noon of the day vaginal plug was detected was considered embryonic day 0.5 (E0.5), and exposure ended at E17.5, at which point pregnant mice were euthanized by overdose with tribromoethanol (intraperitoneal injection, i.p., 650 mg/kg). Seven pregnant dams were used for this E17.5 study, 3 FA and 4 DE. The uterus of each dam was collected and a visual inspection was conducted to detect evidence of embryo resorption. Following removal of the uterus, a gravimetric analysis was performed on the following tissues: uterus, individual whole embryos (including placenta, amnion, fetus, and membranes), individual placentas, and individual fetuses. Three placentas and fetuses from each dam were fixed in 10% neutral buffered formalin and processed with paraffin for further histological analysis.

### Tissue Histology, Placental 3-nitrotyrosine Immunofluorescence, and α-CD45 Immunohistochemistry

To assess the placental pathology, tissues were collected, fixed in 10% neutral buffered formalin and processed following standard protocols. Seven-micron thick sections from both sagittal and *en face* orientations of the placenta were de-paraffinized in xylene, and hydrated using 100%, 95%, and 70% ethanol incubation steps prior to incubation in distilled H2O and subsequent staining with hematoxylin and eosin (H&E). Following staining, sections were mounted with Permount Mounting Medium (Fisher Scientific, Hampton, NH, USA) and evaluated by a veterinary pathologist unaware of treatment status.

3-nitrotyrosine (3-NT) immunofluorescence was performed as previously described, with modifications [33]. Seven-micron thick sagittal sections of the placenta were processed as described above. Sections were blocked in 10% goat serum in PBS at room temperature for 2 hrs, and then incubated at 4°C in 10% goat serum with primary antibody directed against 3-nitrotyrosine (3-NT)(1∶200 dilution; Millipore 06–284, rabbit IgG). Following overnight 3-NT antibody incubation, slides were rinsed with PBS, and then incubated in the dark for 1 hour with goat anti rabbit Alexa 546 secondary antibody (1∶400 dilution). Slides were subsequently rinsed with PBS, and then fixed with 10% neutral buffered formalin for 5 minutes to preserve secondary stain. Slides were rinsed with PBS, and then mounted using Vectashield, mounting medium for fluorescence with DAPI (Vector Laboratories, Inc., Burlingame, CA, USA). Fluorescence images were captured with a Nikon Eclipse E400 microscope equipped with a TRITC filter (Nikon, Tokyo, Japan), a Mercury-100W lamp (Chiu Technical Corp., Kings Park, NY, USA) and a Nikon Digital Sight (Nikon, Tokyo, Japan) camera. Two sections from each placenta were prepared following this procedure, and 3 pictures under a 20X objective were taken from each section for a total of 6 pictures per placenta. Following background subtraction, number of pixels that are 3-NT positive were quantified by Image J (NIH, Bethesda, MD, USA), and average positive 3-NT staining from 3 placentas for each dam were compared.

To assess inflammatory cell infiltration into the placenta, α-CD45 immunohistochemistry was conducted on seven micron sagittal sections using a Diaminobenzidine (DAB) histochemistry kit with streptavidin-HRP (Cat. # D22187, Life Technologies, Carlsbad, CA, USA) following the manufacturer’s protocol. Rat α-mouse CD45 IgG (1∶50 diluation) (Mat. # 553076, BD Pharmingen, Franklin Lakes, NJ, USA) was used as a primary, and biotinylated α-rat IgG raised in goat (1∶100) was used as a secondary (BA-9400, Vector Labs, Burlingame, CA, USA). Following de-paraffinization and hydration steps, placental sections underwent a 30-minute antigen revealing step with 0.1% Triton X-100 in 0.1% sodium citrate. Following DAB treatment and completion of IHC staining, sections were dehydrated with serial ethanol rinses, incubated in xylene, and mounted with permount mounting medium.

### Assessment of Adult Endpoints Following in Utero Exposure to DE

To examine the effects of *in utero* DE exposure on adult endpoints, a total of 9 female mice became pregnant, 4 were exposed to FA and 5 were exposed to DE. Female mice exposed to DE throughout pregnancy were transferred to FA at the end of exposure on E17.5. Birth was observed to occur between E18.5 and E19.5. All offspring from these 9 litters were raised in FA conditions and were weaned at postnatal day (PND) 21. A total of 26 male offspring were used for this study, 12 FA and 14 DE. At 10 weeks of age, male offspring were transferred from the Northlake facility to the UW Medicine South Lake Union (SLU) SPF vivarium, where mice underwent blood pressure, body weight, echocardiographic assessment, and surgery. Male mice were chosen for this study to eliminate the effect of cyclic hormonal variation.

### Blood Pressure Measurement by Tail Cuff

Blood pressure was measured in male mice by use of the CODA 6 tail-cuff blood pressure system (Kent Scientific, Torrington, CT, USA). Prior to blood pressure measurement, mice were acclimated to restraint by following a 7-day training protocol. Each day, male mice were placed into a plastic restraining tube used for tail-cuff and blood pressure measurement for increasing amounts of time, beginning at 5 min/day. At the end of the 7-day period, mice were accustomed to spending 45 min restrained in individual restraining tubes without overt discomfort. Upon blood pressure measurement, mice were restrained and warm water-bags, monitored at a temperature between 35–37°C, were placed on top and around mice to keep temperature stable. Tails of mice were fitted with CODA O-Cuff and VPR-Cuffs according to the manufacturer’s protocol to ensure proper BP measurements. Settings of the tail-cuff were set to measure BP 10 times without recording data, followed by 10 times while recording data. This procedure was practiced 1X without data collection, and a second time the following day with complete data collection. The average of the second 10X data measurements on the second day were used for BP measurements.

### Echocardiography and Transverse Aortic Constriction Surgeries

Upon transfer of male offspring to the SLU SPF vivarium, mice underwent baseline echocardiographic assessment at 11–12 weeks of age. Under 0.5% isoflurane anesthesia, cardiac size and function were assessed using a Visual Sonics (Toronto, Canada) VEVO 770 system equipped with a 707B scan head as previously described [34], [35]. When the heart rate of the mouse had returned to normal following anesthesia (>520 bpm), parasternal short axis views were obtained under M-mode. Cine loops collected from M-mode views were analyzed for anterior and posterior LV wall thickness as well as LV internal diameter at diastole and systole. Percentage fractional shortening (%FS) was calculated from Visual Sonics Standard Measurements and Calculations. One week after baseline echocardiographic assessment was completed, male mice were randomly assigned to either transverse aortic constriction (TAC) or sham surgeries. For surgeries, male mice between 12 and 14 weeks of age were anesthetized using ketamine (130 mg/kg i.p.) and xylazine (8.8 mg/kg i.p.) and subjected to transverse aortic constriction using a 27-gauge needle as described [28], [34], [35]. To measure cardiac response to surgery, echocardiographic assessments were completed for each mouse at 3 weeks post surgery. All echocardiographic measurements were performed by a blinded observer. Consistent with our previous report [28], within 17 mice subjected to TAC surgery, we observed surgery associated death, as defined by death within 72 hours of surgery, in 4/17 (23.5%) of mice. The rate of this mortality was 1/8 (12.5%) in FA mice, and 3/9 (33%) in *in utero* DE exposed mice. Although this mortality rate appeared to be elevated in *in utero* DE exposed mice, this trend did not reach statistical significance by χ^2^ test.

### Necropsy, Gravimetric Tissue Analysis, and Myocardial Histology for Fibrosis

Three weeks after surgery, mice were sacrificed by overdose with tribromoethanol (i.p. 650 mg/kg) followed by exsanguination. Following sacrifice, tissue weights (ventricle weight, lung weight, liver weight, kidney weight, spleen weight, kidney weights, brain weight) and right tibia length were recorded. Tissues were either stored for histology or snap frozen in liquid nitrogen for subsequent biochemical analysis. Ventricles were cut in half along the sagittal axis to expose a two-chamber view. The first half of the ventricle was placed into formalin for fixation, while the second half was frozen in liquid nitrogen. Fixed ventricles were processed and embedded in paraffin as described above. Seven-micron thick sections were made of the heart, and Masson’s Trichrome staining was performed using standard techniques. Extent of myocardial fibrosis was determined by Masson’s Trichrome stain where percentage of blue stain was quantified over total tissue area from 4X images of the left ventricle free wall using NIH Image J (Bethesda, MD, USA) as previously done [28], [34]. Using NIH Image J, low power images of the LV free wall were assessed in a blinded fashion. The border of the myocardium was traced manually to get a baseline area, then the area that would be considered ‘highly fibrotic’, where there is clear evidence of fibroblast collagen deposition and blue staining, is manually traced to get a subsequent area. The area of the myocardium that would be ‘highly fibrotic’ is expressed as a ‘percentage highly fibrotic area’. Images of representative fibrotic regions were selected for presentation. To assess individual cardiomyocyte area, three 40X images of the LV free wall were taken from non-fibrotic areas of each heart. The individual cross-sectional cell area was quantified using NIH Image J, where the areas of 100 cardiomyocytes were manually traced (∼33 randomly selected cells per 40X image) and averaged for each section as described previously [34]. Of 100 cardiomyocytes within the LV, area is averaged to get a single value that represents average myocyte area per heart. We then averaged cardiomyocyte area within exposure and surgery groups to assess individual cardiomyocyte hypertrophy.

### Statistical Analysis

Statistical analyses were performed using GraphPad Prism 5 for Microsoft OS X (GraphPad Software, Inc.; San Diego, CA, USA). Differences between two groups were determined by Student’s T-Test using an α-value of 0.05. To test the effect of heart failure stimulation and exposure, a two-way ANOVA was used followed by a Bonferroni post hoc comparison to test differences between means. All error bars in figures represent mean ± standard error of the mean; *, **, *** represent significant differences with p<0.05, p<0.01, and p<0.001 respectively.

## Results

### In Utero DE Exposure Increases Embryo Resorption, Placental Inflammation, and Placental Oxidative Stress

**Figure 1:**
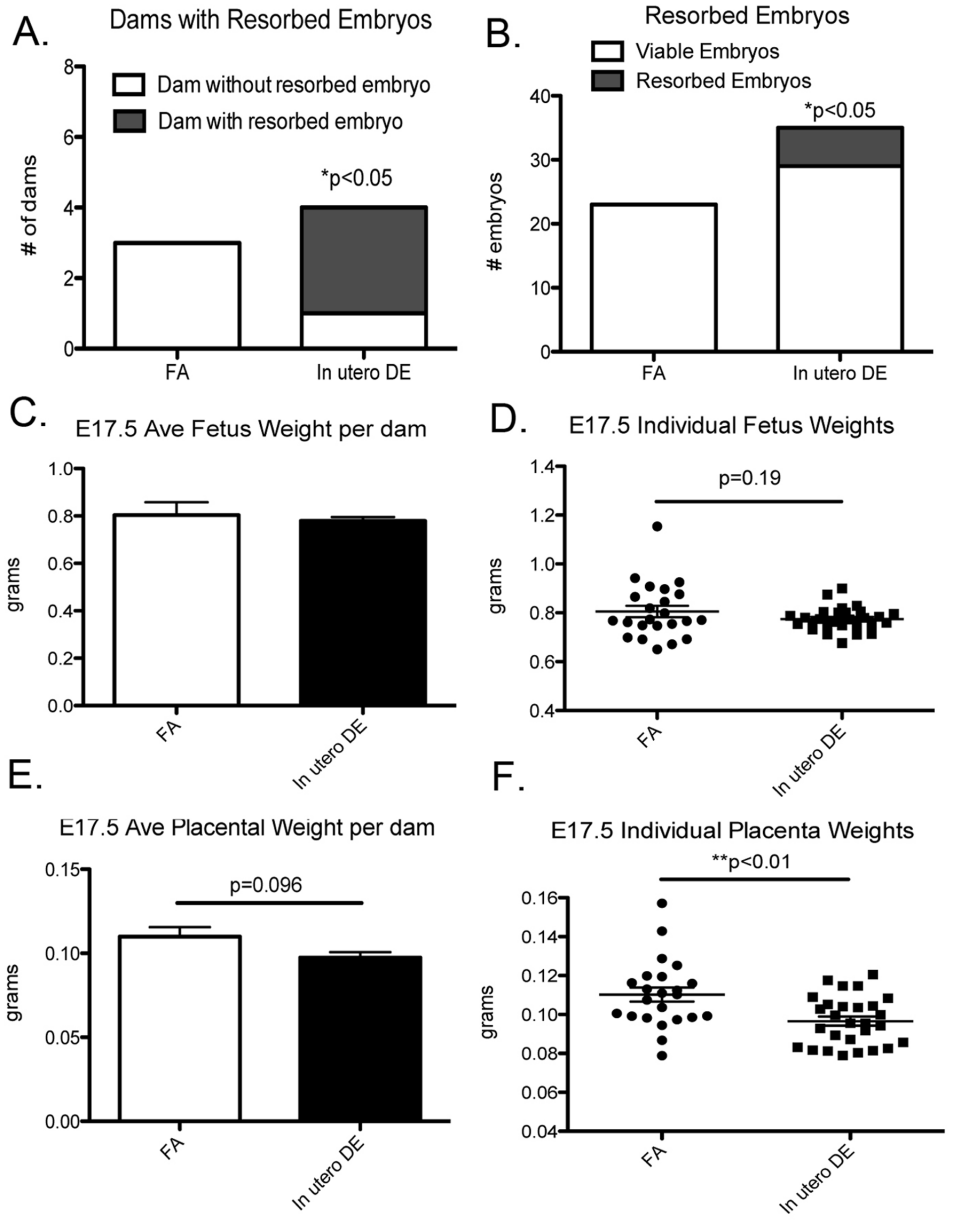
Embryonic day 17.5 (E17.5) embryo collection in FA and DE exposed dams. (A) Number of dams with resorbed embryos and (B) number of embryos resorbed vs number of viable embryos in FA and DE exposed dams. (C) Average fetus weight per dam and (D) individual fetus weights. (E) Average placental weight per dam and (F) individual placental weights. FA dams (n = 3), DE dams (n = 4).

To investigate the effect of *in utero* DE exposure on embryonic development, we exposed C57BL6/J mice to DE beginning on E0.5, 6 hrs/day, 5 days a week (M-F) until E17.5. At E17.5 dams were sacrificed. Uterine tissues were examined for evidence of embryo resorption, and weights were collected for each individual fetus and placenta. Interestingly, we observed the presence of resorbed embryos to be significantly increased within the dams exposed to DE compared to dams exposed to FA (Figure 1A and B). In a further assessment of fetal and placental weight from viable embryos, we did not observe DE to affect fetal weight (Figure 1C and D), but we did find evidence that DE seemed to promote a reduction in placental weight (Figure 1E and F). Although statistical significance is not reached when the average placental weight per dam is compared between FA and DE (n = 3 vs n = 4, respectively, p = 0.096), when statistical analysis is compared from each individual placenta (n = 23 vs n = 28), a strong statistical difference is achieved (**p<0.01).

**Figure 2:**
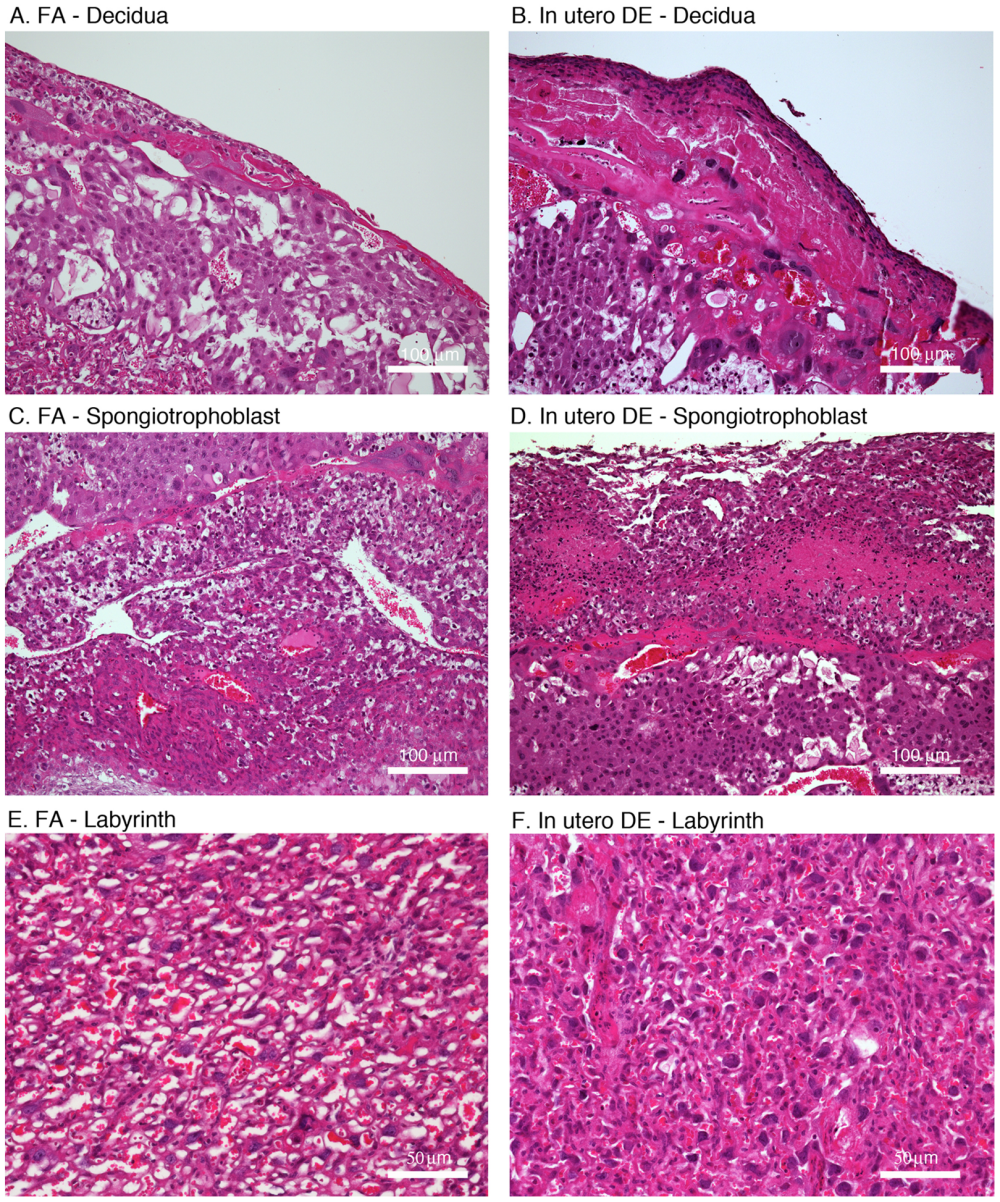
Images of placental cross sections stained with hematoxylin and eosin. (A) FA – Decidua layer, (B) DE – Decidua layer, excessive densely packed fibrin and enmeshed red cells and nuclear debris deep in the spongiotrophoblast layer, (C) FA – spongiotrophoblast layer, (D) DE – spongiotrophoblast layer, focal-extensive area of necrosis, congestion and hemorrhage at the labyrinth/spongiotrophoblast interface, (E) FA – labyrinth layer, (F) DE – labyrinth layer, increased stromal density and compaction of the vascular spaces. Scale bars = panels A–D, 100 µm; panels E and F, 50 µm.

We next assessed the general placental pathology from FA and DE dams by H&E staining. Relative to FA control placentas, those from DE dams showed evidence of increased necrosis, focal congestion, and hemorrhage at the labyrinth/spongiotrophoblast interface (Figure 2A–D). In addition, *in utero* DE exposure was associated with diffuse to focally-extensive compaction and/or increased stromal density of the highly vascularized labyrinth, potentially diminishing vascular space available for blood flow (Figure 2E and F).

**Figure 3:**
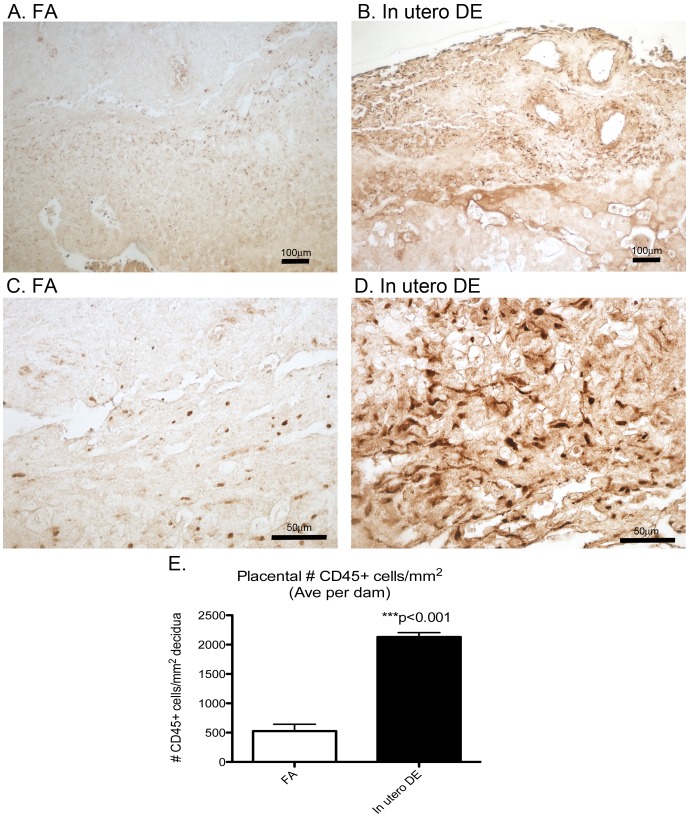
Images of placental cross-sections with immunohistochemistry staining against CD45 in FA (A and C) and *in utero* DE (B and D). Number of CD45+ cells normalized to decidua cross sectional area is quantified (E). FA dams (n = 3), FA placentas (n = 3 per dam), DE dams (n = 4), DE placentas (n = 3 per dam). Scale bars = 100 µm (panels A and B) and 50 µm (panels C and D).

To investigate whether the observed placental pathologies associated with DE exposure are associated with inflammatory cell infiltration, immunohistochemistry against CD45 was performed. CD45+ cells can be observed within the decidua layer in placentas from both FA and DE dams (Figure 3), but the number of cells was increased in placentas exposed to DE (Figure 3B and D). Quantification of the number of CD45+ cells within the decidua layer reveals a significant increase in CD45+ cellular infiltration in the *in utero* DE exposed placentas (Figure 3E).

**Figure 4:**
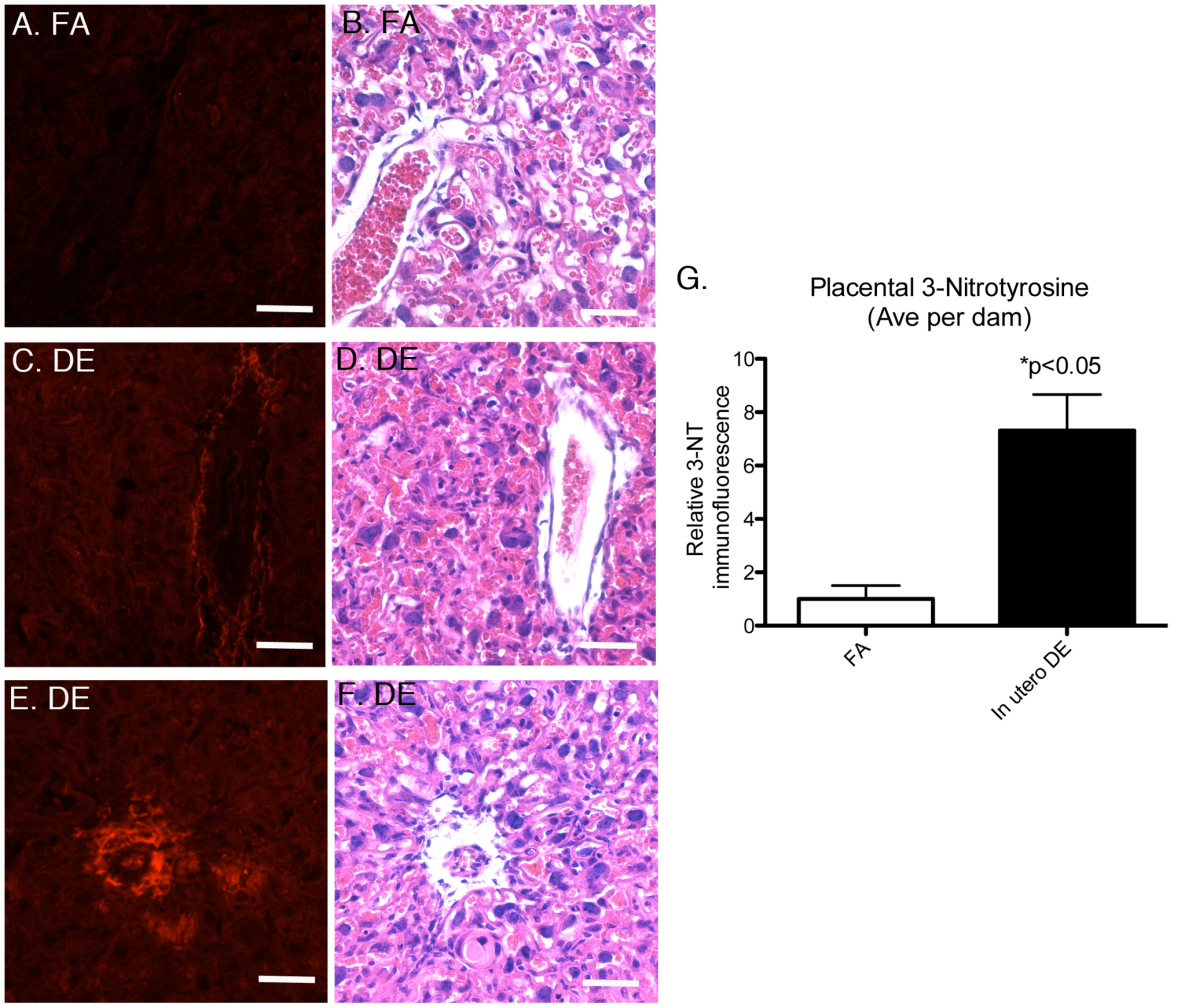
Placental 3-nitrotyrosine (3-NT) staining by immunofluorescence and adjacent H&E stained sections. Representative images from sagittal cross-section from (A and B) FA exposed dam, (C and D) DE exposed dam showing perivascular 3-NT staining, (E and F) DE exposed dam showing perivascular 3-NT staining, and (G) quantification of relative 3-NT fluorescence. FA dams (n = 3), FA placentas (n = 3 per dam), DE dams (n = 4), DE placentas (n = 3 per dam). Scale bars = 50 µm.

To investigate if there is any evidence of DE-induced placental oxidative stress, we measured 3-nitrotyrosine (3-NT) protein modification, a robust marker of oxidative/nitrosative injury, by immunofluorescence in sagittal cross-sections of the placenta. We did not observe any clear evidence of overt 3-NT protein modification in the placental cross-sections from FA exposed dams (Figure 4A). In contrast, 3-NT immunofluorescence from placental cross-sections of DE exposed dams revealed clear evidence of elevated 3-NT protein modification, predominantly within perivascular regions (Figure 4C–F) in the fetal labyrinth layer. This spatial distribution of 3-NT immunofluorescence suggests that *in utero* DE promotes vascular oxidative stress.

### In Utero DE Exposure Increases Body Weight and Alters Blood Pressure in Adult Male Mice

**Figure 5:**
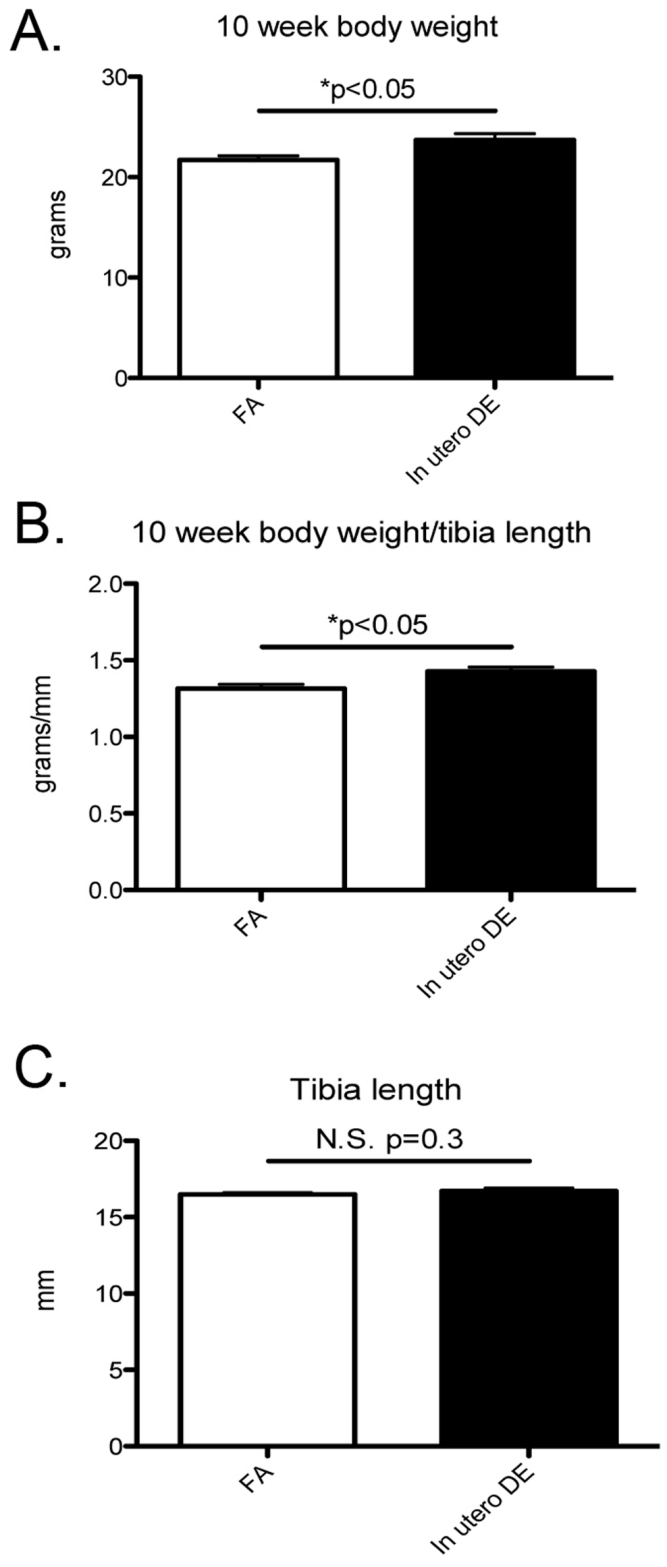
Effects of *in utero* DE exposure on adult body weight. (A) Male 10-week body weight, (B) body weight normalized to tibia length, and (C) tibia length. FA males (n = 10), DE males (n = 9).

Since *in utero* DE exposure is associated with significant placental pathology, we investigated whether *in utero* exposure to DE promotes long-term effects on adult body weight and blood pressure. We observed 10-week body weight to be elevated in male mice exposed to *in utero* DE (Figure 5A). In addition, to control for effects on body habitus rather than animal size, 10-week body weight was normalized to tibia length, a measure collected at necropsy at the end of the study. Body weight normalized to tibia length remained elevated in male mice exposed to DE *in utero* (Figure 5B), whereas no significant differences were observed when comparing tibia lengths (Figure 5C), indicating that effects on body weight are mediated through effects on body habitus.

**Figure 6:**
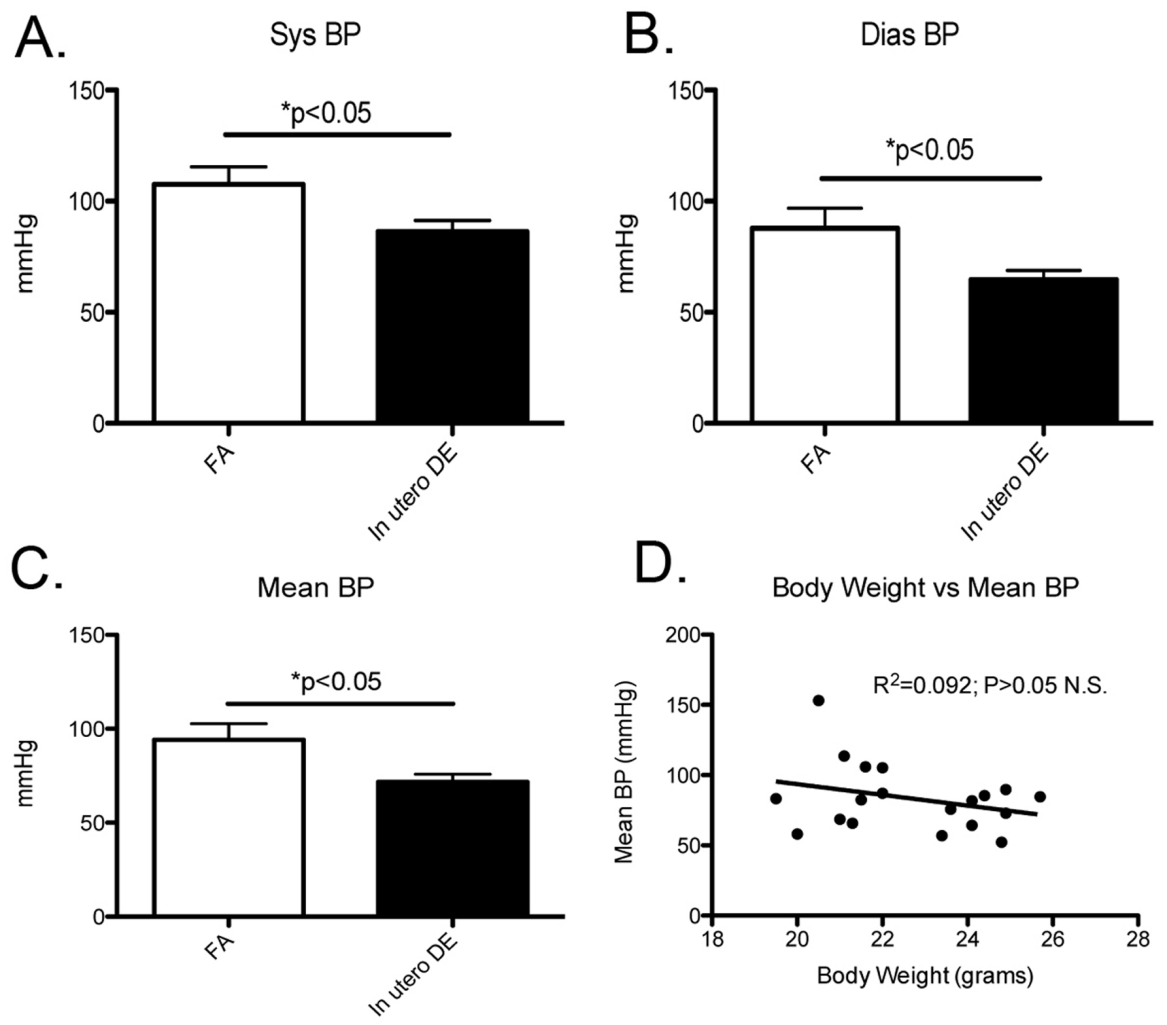
Systolic (A), diastolic (B), and mean (C) arterial pressure as measured by tail-cuff in 10-week old male mice. (D) Linear regression between body weight and mean BP. FA males (n = 10), DE males (n = 9).

As adverse intrauterine conditions have been associated with elevations in adult blood pressure [21], we measured blood pressure by tail-cuff in the same cohorts of male mice that demonstrated an increase in body weight. Interestingly, in contrast to our hypothesis, we observed systolic (SBP), diastolic (DBP), as well as mean arterial blood pressure (MAP) to be significantly decreased in 10-week old male mice exposed to DE *in utero* (Figure 6A–C). To test if these observed decreases in BP were associated with our observed increases in body weight, we performed a linear regression between body weight and MAP (Figure 6D). We did not observe any significant linear relationship between MAP and body weight.

### In Utero DE Exposure does not Alter Adult Baseline Cardiac Function but Increases Susceptibility to Pressure Overload-induced Heart Failure

**Figure 7:**
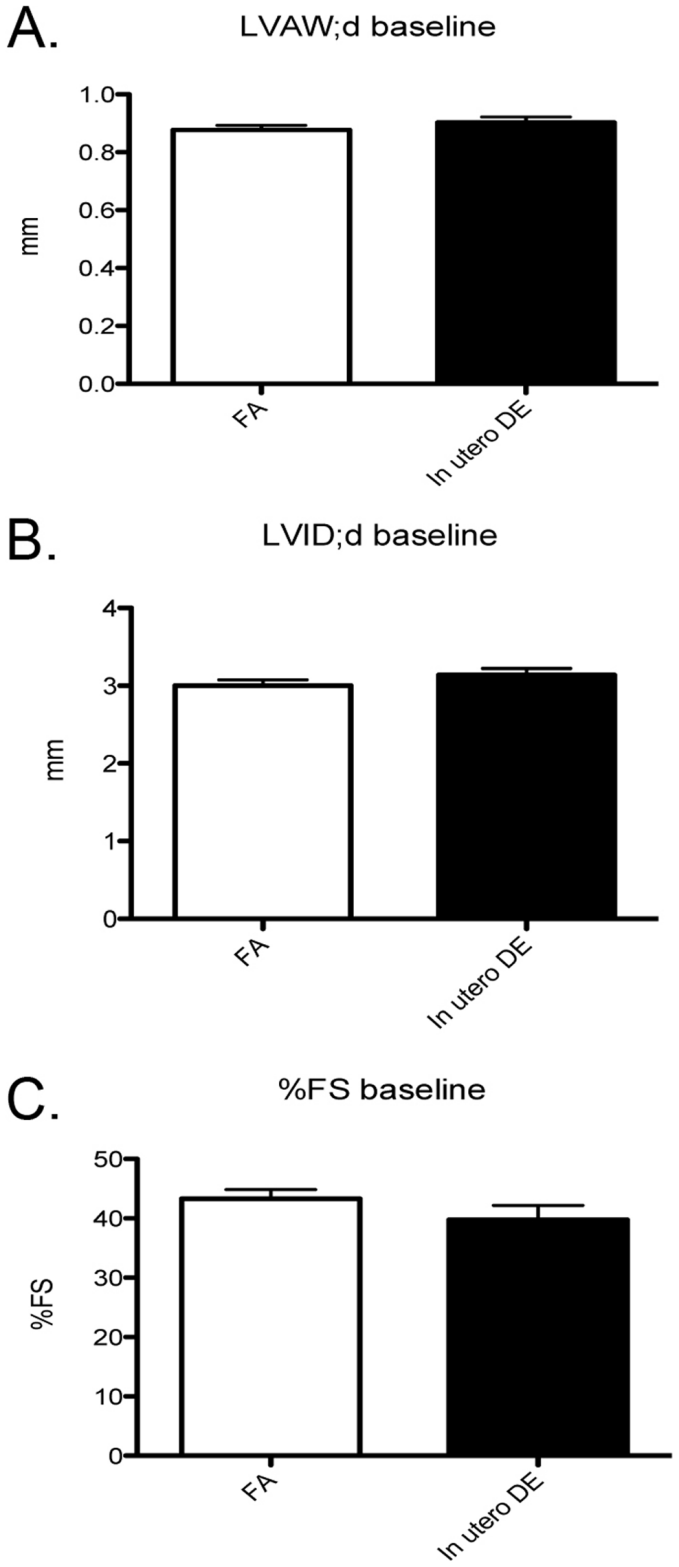
Baseline left ventricular wall thickness and contractile function in 12 week old male mice as measured by echocardiography. (A) Left ventricular anterior wall thickness at diastole, (B) left ventricular internal diameter at diastole, (C) percentage fractional shortening. FA males (n = 10), DE males (n = 9).

At 12-weeks of age, male mice underwent a baseline echocardiographic assessment. We did not observe *in utero* exposure to DE to have any effect on baseline ventricular wall thickness, chamber dimension, and contractile function (Figure 7A–C).

**Figure 8:**
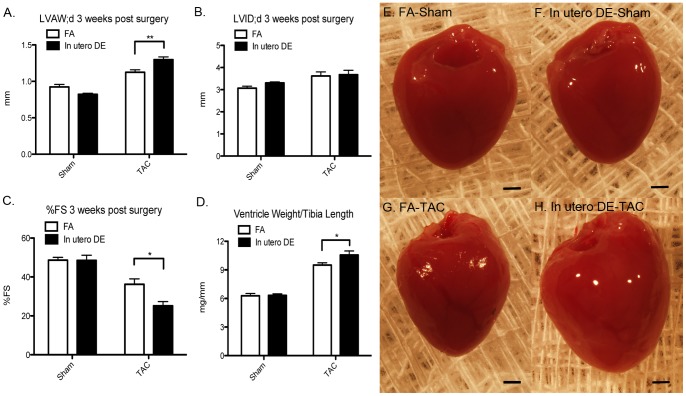
Three weeks following sham or transverse aortic constriction (TAC) surgeries to stimulate pressure overload-induced heart failure, (A) left ventricular anterior wall thickness at diastole, (B) left ventricular interior wall thickness at diastole, (C) percentage fractional shortening, and (D) ventricle weight normalized to tibia length at necropsy. (E–H) Representative images of ventricles at necropsy. FA sham (n = 4), FA TAC (n = 7), DE sham (n = 5), DE TAC (n = 6). Scale bars = 1 mm.

Between 13 and 14 weeks of age, male mice were randomly assigned to undergo sham or transverse aortic constriction (TAC) surgery to promote pressure overload-induced heart failure. At 3 weeks post TAC surgery, an echocardiographic assessment was performed to determine the effect of TAC surgery on cardiac size, dimension, and function, followed by euthanasia and gravimetric analysis (Figure 8). TAC surgery induced cardiac hypertrophy and systolic dysfunction in both FA and DE mice, but mice exposed to DE *in utero* have significantly increased ventricular wall thickness (Figure 8A) in the absence of any change in LV internal diameter (Figure 8B), decreased fractional shortening (Figure 8C), and increased ventricle weight normalized to tibia length (Figure 8D–H).

### In Utero DE Exposure Promotes Myocardial Fibrosis but not Cardiac Myocyte Hypertrophy Following Pressure Overload

**Figure 9:**
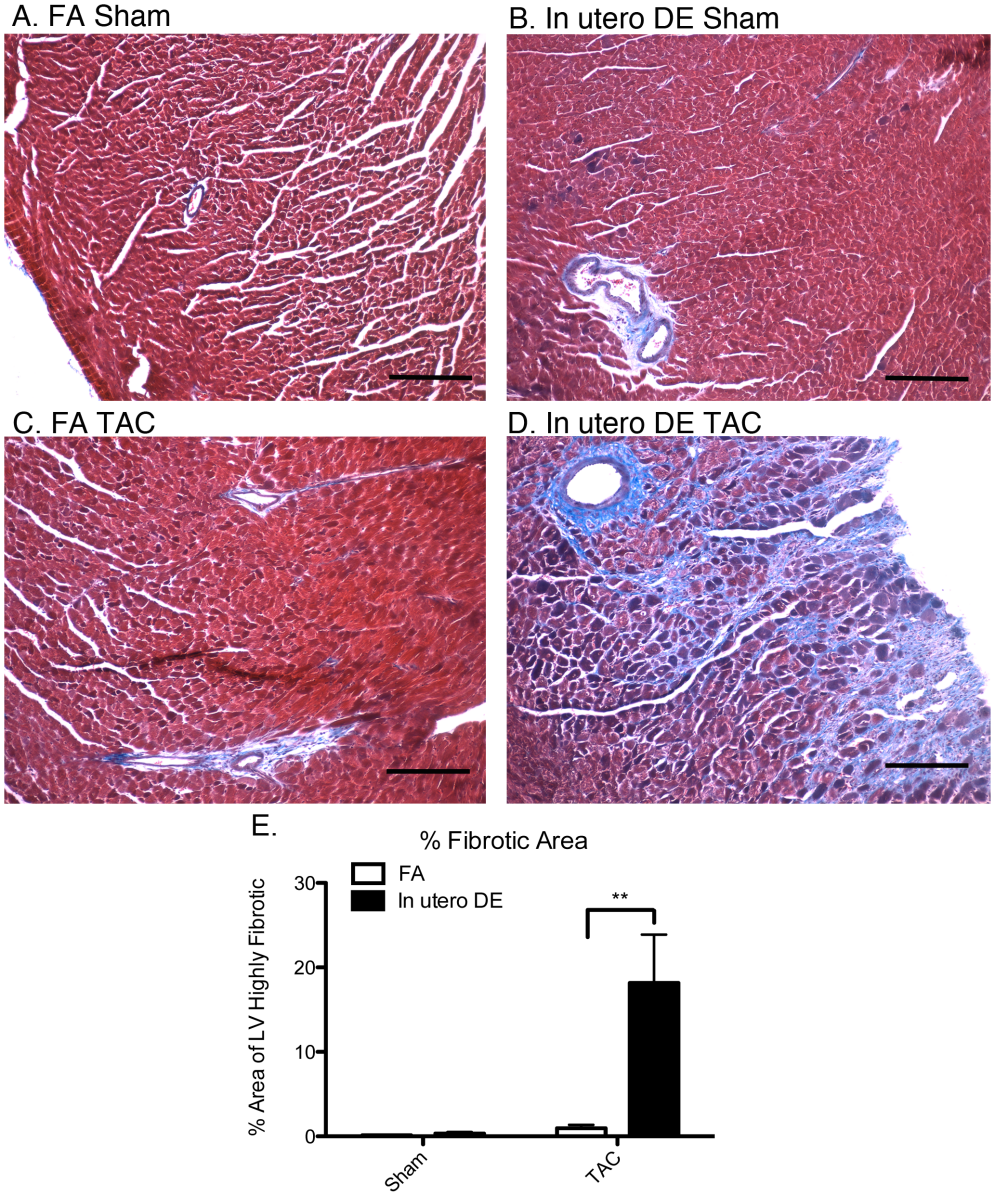
Assessment of myocardial fibrosis in the left ventricle wall of FA Sham (A), *in utero* DE Sham (B), FA TAC (C), and *in utero* DE TAC (D) mice. Blue staining indicates fibrotic regions. Percentage of LV area that is highly fibrotic was quantified (E). FA sham (n = 4), FA TAC (n = 6), DE sham (n = 5), DE TAC (n = 6). Scale bars = 200 µm.

To assess whether *in utero* exposure to DE promotes myocardial fibrosis following sham or TAC surgeries, we performed Masson’s Trichrome staining on sagittal ventricle cross sections and assessed area of highly fibrotic regions as previously described [28], [34]. We did not observe any evidence of fibrosis in the hearts from FA-Sham mice (Figure 9A), and only mild perivascular fibrosis in FA-TAC and in utero DE-Sham hearts (Figure 9B and C). In contrast, the hearts from *in utero* DE-TAC mice showed extensive perivascular and interstitial fibrosis distributed diffusely across the left ventricular free wall. Quantification of fibrotic area demonstrated that fibrosis within the *in utero* DE-TAC mice was significantly greater than FA-TAC mice (FA-TAC n = 7, *in utero* DE-TAC n = 6)(**p<0.01)(Figure 9E).

**Figure 10:**
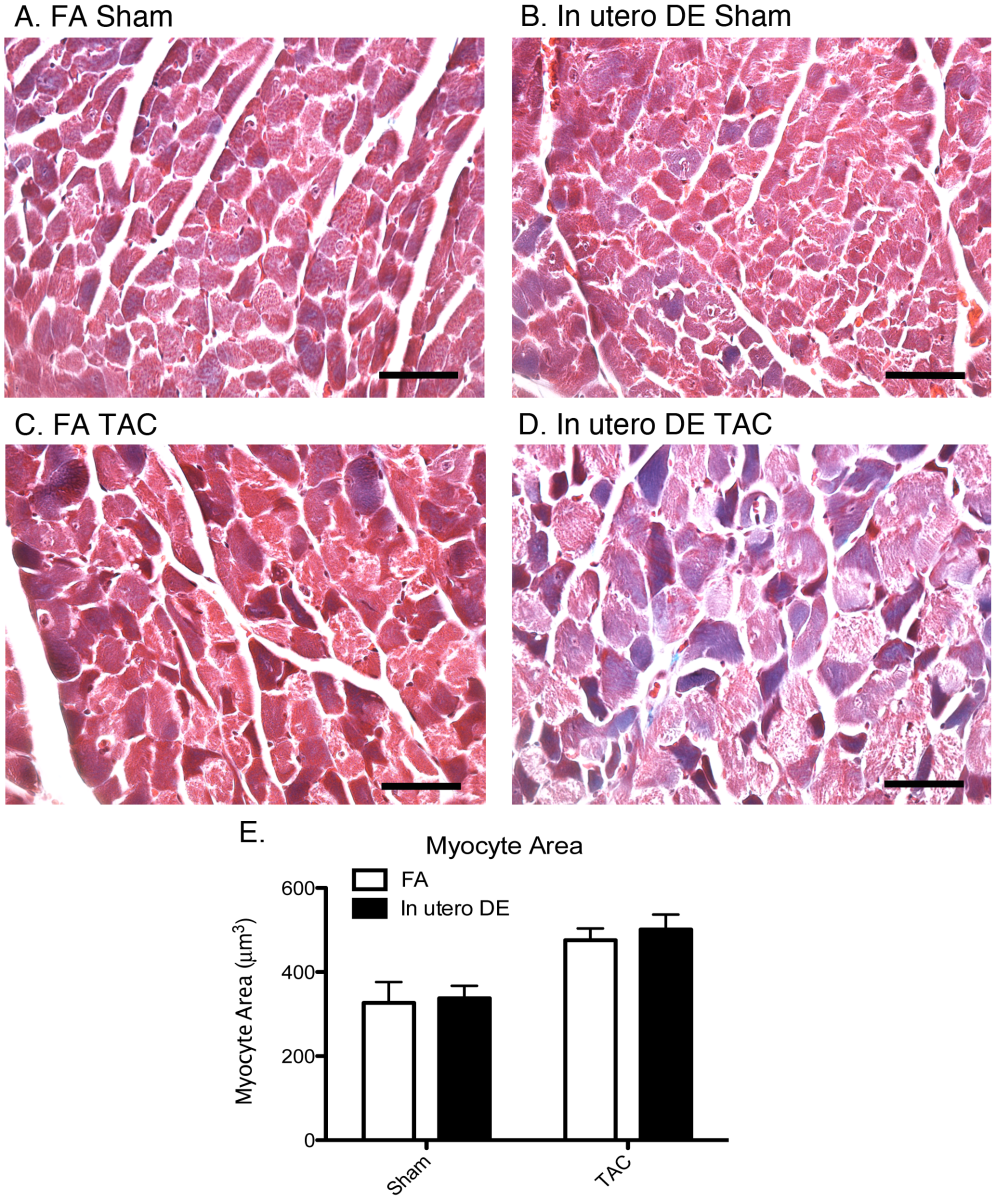
Assessment of individual cardiomyocyte hypertrophy in the left ventricle wall of FA Sham (A), *in utero* DE Sham (B), FA TAC (C), and *in utero* DE TAC (D) mice. Average myocyte area was quantified (E). FA sham (n = 4), FA TAC (n = 6), DE sham (n = 5), DE TAC (n = 6). Scale bars = 50 µm.

To assess whether the observed effect of *in utero* DE exposure on heart weight is mediated at the myocyte level, we assessed the extent of individual cardiomyocyte hypertrophy by measuring individual myocyte cross-sectional area in exposed sham and TAC mice. We observed TAC to significantly increase individual cardiomyocyte cross-sectional area in both FA and *in utero* DE exposed mice (Figure 10) but did not observe an additive effect of *in utero* exposure to DE (Figure 10E).

## Discussion

In this report, we provide evidence that *in utero* exposure to diesel exhaust air pollution (≈300 µg/m^3^ PM_2.5_, 6 hrs/day, 5 days/week, a 53 µg/m^3^/hr PM_2.5_ time weighted average) promotes significant placental injury, manifested by hemorrhage, vascular compromise, focal necrosis, embryo resorption, inflammation, and oxidative stress. Surviving embryos develop increased body weight, altered blood pressure, and increased susceptibility to pressure overload-induced heart failure as adults. In combination, these data provide strong evidence that *in utero* exposure to fine particular air pollution may have significant effects on adult susceptibility to cardiovascular disease and heart failure. Although we have previously reported that combined *in utero* and early life exposure to DE can promote adult susceptibility to heart failure [28], these data reported here extend our earlier findings by demonstrating that *in utero* exposure to DE alone is sufficient to affect adult susceptibility to heart failure, supporting a fetal origin for this susceptibility to adult disease, through a mechanism that involves placental injury.

Placental insufficiency results from placental injury and has been reported to promote fetal hypoglycemia, hypoinsulinemia, acidosis, hypoxia, and decreased branched chain amino acid transfer [36], [37], [38], [39], [40], [41] that can result in ‘reprogramming’ events that alter metabolic, cellular, and physiological function throughout life [39], [41], [42], [43], [44], [45], [46]. Importantly, it has been suggested that many of these altered metabolic states are due to stable changes in gene expression resulting from epigenetic modifications such as DNA and histone methylation [39], [43]. Fetal hypoxia, a potential complication of placental injury, has been reported to promote fetal inflammation and oxidative stress, resulting in elevated DNA methylation of the promoter region of PKCε, predisposing adult myocardium to ischemia-reperfusion injury [47], [48]. Our findings are consistent with such a model, as the increased body weight to tibia length, decreased blood pressure, and increased susceptibility to heart failure in male mice exposed *in utero* is consistent with metabolic and physiologic reprogramming. Whether our effect is due to epigenetic reprogramming events that occur during *in utero* development is currently under investigation.

*In utero* exposure to other toxic agents have also been reported to promote adult cardiovascular disease. *In utero* exposure to AZT and 3TC reportedly leads to heart failure in adult female mice associated with increased cardiac mitochondrial DNA content and altered mitochondrial and myofibril ultrastructure [49], [50]. *In utero* exposure to caffeine also reportedly predisposes to increased adult body fat composition and adult heart failure, possibly through inhibition of A1AR and HIF1α activity [51]. *In utero* exposure to cocaine has been shown to promote fetal cardiac myocyte apoptosis, adult susceptibility to ischemia-reperfusion and adult hypertension [52], [53], [54]. In our study, we have not yet assessed mitochondrial or myofibril ultrastructure, myocardial apoptosis or the potential role of A1AR or HIF1α. It is important to note, however, that antiretroviral drugs and caffeine exposure both result in baseline ventricular dysfunction, which we do not observe in our study. Cocaine exposure results in adult hypertension, while we observe reduced blood pressure, suggesting independent pathophysiological mechanisms. Reduced blood pressure has also been observed in adult rats with diet-induced metabolic syndrome after acute exposure to PM_2.5_ and ozone, through a presumed autonomic mechanism [55]. Further investigation is required to determine whether there may be overlapping toxicological mechanisms in common between these reports and our study.

In our study, we tested the effect of DE exposure, at ≈300 µg/m^3^ PM_2.5_, 6 hours/day, 5 days/week. A 300 µg/m^3^ PM_2.5_ concentration for this exposure paradigm equates to a time weighted average of 53 µg/m^3^/hr PM_2.5_. This hourly exposure is significantly higher than the current U.S. EPA clean air standard of 12 µg/m^3^ PM_2.5_ per year. We chose to test this DE concentration as it is consistent with other investigations on DE and vascular function [56], [57], [58], and it is very relevant to population level exposures in highly polluted urban areas where the level of exposure can approach 500 µg/m^3^ at any given time [59].

The relevance of our findings to human populations remains to be determined. A recent meta-analysis of PM exposure and birthweight observed PM_2.5_ and PM_10_ exposure to be associated with reduced birthweight [17] at concentrations observed in developed countries. In addition, PM_2.5_ exposure during pregnancy has been associated with decreased placental mitochondrial DNA content (a marker of mitochondrial oxidative stress) [60] and placental DNA hypomethylation [61]. These observations indicate that human exposure to PM_2.5_, at concentrations relevant to those living in developed countries, directly impact the developing fetus, and likely through a placental insufficiency mechanism. These findings suggest that effects on adult human cardiovascular disease susceptibility are plausible, although further epidemiologic and clinical studies will be necessary to make this determination.

The mechanism by which inhaled PM can elicit placental inflammation and oxidative stress remains unclear. It has been reported that inhaled particles can elicit systemic inflammation, and *in vitro* models have shown that soluble factors released from diesel exhaust particulate exposed macrophages are able to rapidly incite vascular endothelial inflammation and oxidative stress [62], [63]. In addition, it has been observed that human plasma collected from individuals exposed to DE for only short periods of time (1 hr) is proinflammatory to endothelial cells *in vitro* [64], further suggesting that soluble, proinflammatory mediators are circulating in the blood following the inhalation of DE. In our system, it remains to be determined whether there are maternal circulating proinflammatory mediators that are responsible for our observed effects. Although it is possible that circulating factors are causal to these effects, we cannot exclude the possibility that maternal DE exposure promotes the activation of neuronal transient receptor potential (TRP) channels that line the airway and which have been observed to be activated by DE and promote systemic vascular and cardiac effects [65], [66]. TRP channels are highly expressed within female reproductive organs and the placenta, and their activation has been suggested to play important roles in placental development and regulating the feto-maternal interface [67]. If DE exposure can result in systemic activation of TRP channels, it is possible that placental TRP channels are also activated and may mediate our observed effects. This possibility has yet to be investigated.

Taking into account the potential for circulating factors as well as neuronal TRP activation in mediating these systemic effects, we observed increased inflammatory cell infiltration into the maternal decidua layer of the placenta, suggesting inflammation to arise from signals originating from the mother. Interestingly, our observed increase in placental vascular oxidative stress did not overlap with areas of inflammatory cell infiltration and were largely limited to the fetal labyrinth layer. At present, a causal relationship between the observed increases in vascular oxidative stress and inflammatory cell infiltration has not been established and it remains possible that they are unrelated. Regardless of their relationship, they both provide evidence of significant placental injury in response to DE exposure.

## Conclusions

In this report, we demonstrate that *in utero* exposure to diesel exhaust air pollution in mice promotes placental injury, creating long lasting effects on weight gain, blood pressure, and susceptibility to heart failure in surviving embryos. The results from this study raise the question of whether human susceptibility to adult cardiovascular disease may be exacerbated by *in utero* exposure to air pollution and suggest the need for future studies to address this question. Such studies will likely guide future regulatory policies that will address environmental exposures and public health.

## References

[1] Miller KA, Siscovick DS, Sheppard L, Shepherd K, Sullivan JH, et al (2007) Long-term exposure to air pollution and incidence of cardiovascular events in women. N Engl J Med 356: 447–458. 17267905 10.1056/NEJMoa054409

[2] Pope CA, Burnett RT, Thun MJ, Calle EE, Krewski D, et al (2002) Lung cancer, cardiopulmonary mortality, and long-term exposure to fine particulate air pollution. JAMA 287: 1132–1141. 11879110 10.1001/jama.287.9.1132 PMC4037163

[3] Pope CA, Burnett RT, Thurston GD, Thun MJ, Calle EE, et al (2004) Cardiovascular mortality and long-term exposure to particulate air pollution: epidemiological evidence of general pathophysiological pathways of disease. Circulation 109: 71–77. 14676145 10.1161/01.CIR.0000108927.80044.7F

[4] Lim SS, Vos T, Flaxman AD, Danaei G, Shibuya K, et al (2013) A comparative risk assessment of burden of disease and injury attributable to 67 risk factors and risk factor clusters in 21 regions, 1990–2010: a systematic analysis for the Global Burden of Disease Study 2010. Lancet 380: 2224–2260. 10.1016/S0140-6736(12)61766-8 PMC4156511 23245609

[5] Brook RD, Rajagopalan S, Pope CA, Brook JR, Bhatnagar A, et al (2010) Particulate matter air pollution and cardiovascular disease: An update to the scientific statement from the American Heart Association. Circulation 121: 2331–2378. 20458016 10.1161/CIR.0b013e3181dbece1

[6] Bai N, Kido T, Suzuki H, Yang G, Kavanagh TJ, et al (2011) Changes in atherosclerotic plaques induced by inhalation of diesel exhaust. Atherosclerosis 216: 299–306. 21435644 10.1016/j.atherosclerosis.2011.02.019 PMC4631611

[7] Campen MJ, Lund AK, Knuckles TL, Conklin DJ, Bishop B, et al (2010) Inhaled diesel emissions alter atherosclerotic plaque composition in ApoE(−/−) mice. Toxicology and Applied Pharmacology 242: 310–317. 19891982 10.1016/j.taap.2009.10.021 PMC2813974

[8] Quan C, Sun Q, Lippmann M, Chen L-C (2010) Comparative effects of inhaled diesel exhaust and ambient fine particles on inflammation, atherosclerosis, and vascular dysfunction. Inhalation Toxicology 22: 738–753. 20462391 10.3109/08958371003728057 PMC3073494

[9] Sun Q, Wang A, Jin X, Natanzon A, Duquaine D, et al (2005) Long-term air pollution exposure and acceleration of atherosclerosis and vascular inflammation in an animal model. JAMA 294: 3003–3010. 16414948 10.1001/jama.294.23.3003

[10] Kampfrath T, Maiseyeu A, Ying Z, Shah Z, Deiuliis JA, et al.. (2011) Chronic Fine Particulate Matter Exposure Induces Systemic Vascular Dysfunction via NADPH Oxidase and TLR4 Pathways. Circulation Research: 1–29. 10.1161/CIRCRESAHA.110.237560 PMC3085907 21273555

[11] Sun Q, Yue P, Ying Z, Cardounel AJ, Brook RD, et al (2008) Air Pollution Exposure Potentiates Hypertension Through Reactive Oxygen Species-Mediated Activation of Rho/ROCK. Arteriosclerosis, Thrombosis, and Vascular Biology 28: 1760–1766. 10.1161/ATVBAHA.108.166967 PMC2739008 18599801

[12] Wold LE, Ying Z, Hutchinson KR, Velten M, Gorr MW, et al (2012) Cardiovascular remodeling in response to long-term exposure to fine particulate matter air pollution. Circulation Heart failure 5: 452–461. 22661498 10.1161/CIRCHEARTFAILURE.112.966580 PMC3617499

[13] Ying Z, Yue P, Xu X, Zhong M, Sun Q, et al.. (2009) Air pollution and cardiac remodeling: a role for RhoA/Rho-kinase. AJP: Heart and Circulatory Physiology. H1540–H1550. 10.1152/ajpheart.01270.2008 PMC2685340 19286943

[14] Arck PC, Hecher K (2013) Fetomaternal immune cross-talk and its consequences for maternal and offspring's health. Nature medicine 19: 548–556. 10.1038/nm.3160 23652115

[15] Ward JM, Elmore SA, Foley JF (2012) Pathology methods for the evaluation of embryonic and perinatal developmental defects and lethality in genetically engineered mice. Veterinary pathology 49: 71–84. 22146849 10.1177/0300985811429811 PMC13010734

[16] Webster WS, Abela D (2007) The effect of hypoxia in development. Birth defects research Part C, Embryo today : reviews 81: 215–228. 10.1002/bdrc.20102 17963271

[17] Dadvand P, Parker J, Bell ML, Bonzini M, Brauer M, et al (2013) Maternal exposure to particulate air pollution and term birth weight: a multi-country evaluation of effect and heterogeneity. Environmental Health Perspectives 121: 267–373. 23384584 10.1289/ehp.1205575 PMC3621183

[18] Lewtas J (2007) Air pollution combustion emissions: characterization of causative agents and mechanisms associated with cancer, reproductive, and cardiovascular effects. Mutat Res 636: 95–133. 17951105 10.1016/j.mrrev.2007.08.003

[19] Auten RL, Gilmour MI, Krantz QT, Potts EN, Mason SN, et al (2012) Maternal diesel inhalation increases airway hyperreactivity in ozone-exposed offspring. American Journal of Respiratory Cell and Molecular Biology 46: 454–460. 22052876 10.1165/rcmb.2011-0256OC PMC3359947

[20] Bolton JL, Smith SH, Huff NC, Gilmour MI, Foster WM, et al (2012) Prenatal air pollution exposure induces neuroinflammation and predisposes offspring to weight gain in adulthood in a sex-specific manner. The FASEB journal : official publication of the Federation of American Societies for Experimental Biology 26: 4743–4754. 22815382 10.1096/fj.12-210989

[21] Barker DJ, Osmond C, Golding J, Kuh D, Wadsworth ME (1989) Growth in utero, blood pressure in childhood and adult life, and mortality from cardiovascular disease. BMJ (Clinical research ed) 298: 564–567. 10.1136/bmj.298.6673.564 PMC1835925 2495113

[22] Eriksson J, Forsén T, Tuomilehto J, Osmond C, Barker D (2000) Fetal and childhood growth and hypertension in adult life. Hypertension 36: 790–794. 11082144 10.1161/01.hyp.36.5.790

[23] Feldt K, Räikkönen K, Eriksson JG, Andersson S, Osmond C, et al (2007) Cardiovascular reactivity to psychological stressors in late adulthood is predicted by gestational age at birth. Journal of human hypertension 21: 401–410. 17330055 10.1038/sj.jhh.1002176

[24] Godfrey KM, Barker DJ (2001) Fetal programming and adult health. Public health nutrition 4: 611–624. 11683554 10.1079/phn2001145

[25] Law CM, de Swiet M, Osmond C, Fayers PM, Barker DJ, et al (1993) Initiation of hypertension in utero and its amplification throughout life. BMJ (Clinical research ed) 306: 24–27. 10.1136/bmj.306.6869.24 PMC1676382 8435572

[26] Martyn CN, Barker DJ, Jespersen S, Greenwald S, Osmond C, et al (1995) Growth in utero, adult blood pressure, and arterial compliance. British heart journal 73: 116–121. 7696018 10.1136/hrt.73.2.116 PMC483775

[27] Vijayakumar M, Fall CH, Osmond C, Barker DJ (1995) Birth weight, weight at one year, and left ventricular mass in adult life. British heart journal 73: 363–367. 7756071 10.1136/hrt.73.4.363 PMC483831

[28] Weldy CS, Liu Y, Chang Y-C, Medvedev IO, Fox JR, et al (2013) In utero and early life exposure to diesel exhaust air pollution increases adult susceptibility to heart failure in mice. Particle and fibre toxicology 10: 59. 24279743 10.1186/1743-8977-10-59 PMC3902482

[29] Gould T, Larson T, Stewart J, Kaufman JD, Slater D, et al (2008) A controlled inhalation diesel exhaust exposure facility with dynamic feedback control of PM concentration. Inhalation Toxicology 20: 49–52. 18236222 10.1080/08958370701758478

[30] Weldy CS, Luttrell IP, White CC, Morgan-Stevenson V, Cox DP, et al (2013) Glutathione (GSH) and the GSH synthesis gene Gclm modulate plasma redox and vascular responses to acute diesel exhaust inhalation in mice. Inhalation Toxicology 25: 444–454. 23808636 10.3109/08958378.2013.801004 PMC3831526

[31] Yin F, Lawal A, Ricks J, Fox JR, Larson T, et al (2013) Diesel exhaust induces systemic lipid peroxidation and development of dysfunctional pro-oxidant and pro-inflammatory high-density lipoprotein. Arteriosclerosis, Thrombosis, and Vascular Biology 33: 1153–1161. 10.1161/ATVBAHA.112.300552 23559632

[32] Liu Y, Chien W-M, Medvedev IO, Weldy CS, Luchtel DL, et al (2013) Inhalation of diesel exhaust does not exacerbate cardiac hypertrophy or heart failure in two mouse models of cardiac hypertrophy. Particle and fibre toxicology 10: 49. 24093778 10.1186/1743-8977-10-49 PMC3851491

[33] Weldy CS, Luttrell IP, White CC, Morgan-Stevenson V, Bammler TK, et al (2012) Glutathione (GSH) and the GSH synthesis gene Gclm modulate vascular reactivity in mice. Free Radical Biology and Medicine 53: 1264–1278. 22824862 10.1016/j.freeradbiomed.2012.07.006 PMC3625031

[34] Liu Y, Yu M, Wu L, Chin MT (2010) The bHLH transcription factor CHF1/Hey2 regulates susceptibility to apoptosis and heart failure after pressure overload. American journal of physiology Heart and circulatory physiology 298: H2082–2092. 20382855 10.1152/ajpheart.00747.2009 PMC2886641

[35] Yu M, Liu Y, Xiang F, Li Y, Cullen D, et al (2009) CHF1/Hey2 promotes physiological hypertrophy in response to pressure overload through selective repression and activation of specific transcriptional pathways. Omics : a journal of integrative biology 13: 501–511. 20001863 10.1089/omi.2009.0086 PMC3145963

[36] Bussey ME, Finley S, LaBarbera A, Ogata ES (1985) Hypoglycemia in the newborn growth-retarded rat: delayed phosphoenolpyruvate carboxykinase induction despite increased glucagon availability. Pediatric research 19: 363–367. 3889814

[37] Economides DL, Nicolaides KH (1989) Blood glucose and oxygen tension levels in small-for-gestational-age fetuses. American journal of obstetrics and gynecology 160: 385–389. 2916623 10.1016/0002-9378(89)90453-5

[38] Economides DL, Nicolaides KH, Gahl WA, Bernardini I, Bottoms S, et al (1989) Cordocentesis in the diagnosis of intrauterine starvation. American journal of obstetrics and gynecology 161: 1004–1008. 2679098 10.1016/0002-9378(89)90772-2

[39] MacLennan NK, James SJ, Melnyk S, Piroozi A, Jernigan S, et al (2004) Uteroplacental insufficiency alters DNA methylation, one-carbon metabolism, and histone acetylation in IUGR rats. Physiological genomics 18: 43–50. 15084713 10.1152/physiolgenomics.00042.2004

[40] Ogata ES, Bussey ME, Finley S (1986) Altered gas exchange, limited glucose and branched chain amino acids, and hypoinsulinism retard fetal growth in the rat. Metabolism: clinical and experimental 35: 970–977. 3531762 10.1016/0026-0495(86)90064-8

[41] Ogata ES, Bussey ME, LaBarbera A, Finley S (1985) Altered growth, hypoglycemia, hypoalaninemia, and ketonemia in the young rat: postnatal consequences of intrauterine growth retardation. Pediatric research 19: 32–37. 3881726 10.1203/00006450-198501000-00010

[42] Bagley HN, Wang Y, Campbell MS, Yu X, Lane RH, et al (2013) Maternal docosahexaenoic acid increases adiponectin and normalizes IUGR-induced changes in rat adipose deposition. Journal of obesity 2013: 312153. 23533720 10.1155/2013/312153 PMC3606778

[43] Ke X, Lei Q, James SJ, Kelleher SL, Melnyk S, et al (2006) Uteroplacental insufficiency affects epigenetic determinants of chromatin structure in brains of neonatal and juvenile IUGR rats. Physiological genomics 25: 16–28. 16380407 10.1152/physiolgenomics.00093.2005

[44] Lane RH, Kelley DE, Gruetzmacher EM, Devaskar SU (2001) Uteroplacental insufficiency alters hepatic fatty acid-metabolizing enzymes in juvenile and adult rats. American journal of physiology Regulatory, integrative and comparative physiology 280: R183–190. 10.1152/ajpregu.2001.280.1.R183 11124150

[45] Lane RH, Kelley DE, Ritov VH, Tsirka AE, Gruetzmacher EM (2001) Altered expression and function of mitochondrial beta-oxidation enzymes in juvenile intrauterine-growth-retarded rat skeletal muscle. Pediatric research 50: 83–90. 11420423 10.1203/00006450-200107000-00016

[46] Tsirka AE, Gruetzmacher EM, Kelley DE, Ritov VH, Devaskar SU, et al (2001) Myocardial gene expression of glucose transporter 1 and glucose transporter 4 in response to uteroplacental insufficiency in the rat. The Journal of endocrinology 169: 373–380. 11312153 10.1677/joe.0.1690373

[47] Patterson AJ, Chen M, Xue Q, Xiao D, Zhang L (2010) Chronic prenatal hypoxia induces epigenetic programming of PKC{epsilon} gene repression in rat hearts. Circulation Research 107: 365–373. 20538683 10.1161/CIRCRESAHA.110.221259 PMC2919213

[48] Patterson AJ, Xiao D, Xiong F, Dixon B, Zhang L (2012) Hypoxia-derived oxidative stress mediates epigenetic repression of PKCε gene in foetal rat hearts. Cardiovascular Research 93: 302–310. 22139554 10.1093/cvr/cvr322 PMC3258654

[49] Torres SM, Divi RL, Walker DM, McCash CL, Carter MM, et al (2010) In utero exposure of female CD-1 mice to AZT and/or 3TC: II. Persistence of functional alterations in cardiac tissue. Cardiovascular toxicology 10: 87–99. 20155331 10.1007/s12012-010-9065-z PMC3189686

[50] Torres SM, March TH, Carter MM, McCash CL, Seilkop SK, et al (2010) In utero exposure of female CD-1 Mice to AZT and/or 3TC: I. Persistence of microscopic lesions in cardiac tissue. Cardiovascular toxicology 10: 37–50. 20101476 10.1007/s12012-010-9061-3 PMC2867104

[51] Wendler CC, Busovsky-McNeal M, Ghatpande S, Kalinowski A, Russell KS, et al (2009) Embryonic caffeine exposure induces adverse effects in adulthood. The FASEB journal : official publication of the Federation of American Societies for Experimental Biology 23: 1272–1278. 19088180 10.1096/fj.08-124941 PMC2660649

[52] Bae S, Gilbert RD, Ducsay CA, Zhang L (2005) Prenatal cocaine exposure increases heart susceptibility to ischaemia-reperfusion injury in adult male but not female rats. The Journal of Physiology 565: 149–158. 15677681 10.1113/jphysiol.2005.082701 PMC1464496

[53] Bae S, Zhang L (2005) Prenatal cocaine exposure increases apoptosis of neonatal rat heart and heart susceptibility to ischemia-reperfusion injury in 1-month-old rat. British Journal of Pharmacology 144: 900–907. 15685203 10.1038/sj.bjp.0706129 PMC1576080

[54] Xiao D, Yang S, Zhang L (2009) Prenatal cocaine exposure causes sex-dependent impairment in the myogenic reactivity of coronary arteries in adult offspring. Hypertension 54: 1123–1128. 19704103 10.1161/HYPERTENSIONAHA.109.138024 PMC2768326

[55] Wagner JG, Allen K, Yang H-YY, Nan B, Morishita M, et al (2013) Cardiovascular Depression in Rats Exposed to Inhaled Particulate Matter and Ozone: Effects of Diet-Induced Metabolic Syndrome. Environmental Health Perspectives 122: 27–33. 24169565 10.1289/ehp.1307085 PMC3888573

[56] Cherng TW, Campen MJ, Knuckles TL, Gonzalez Bosc L, Kanagy NL (2009) Impairment of coronary endothelial cell ETB receptor function after short-term inhalation exposure to whole diesel emissions. AJP: Regulatory, Integrative and Comparative Physiology 297: R640–R647. 10.1152/ajpregu.90899.2008 PMC2739797 19535675

[57] Cherng TW, Paffett ML, Jackson-Weaver O, Campen MJ, Walker BR, et al (2011) Mechanisms of diesel-induced endothelial nitric oxide synthase dysfunction in coronary arterioles. Environmental Health Perspectives 119: 98–103. 20870565 10.1289/ehp.1002286 PMC3018507

[58] Knuckles TL, Lund AK, Lucas SN, Campen MJ (2008) Diesel exhaust exposure enhances venoconstriction via uncoupling of eNOS. Toxicology and Applied Pharmacology 230: 346–351. 18455212 10.1016/j.taap.2008.03.010

[59] Wang J-F, Hu M-G, Xu C-D, Christakos G, Zhao Y (2013) Estimation of citywide air pollution in Beijing. PloS one 8: e53400. 23320082 10.1371/journal.pone.0053400 PMC3539974

[60] Janssen BG, Munters E, Pieters N, Smeets K, Cox B, et al (2012) Placental mitochondrial DNA content and particulate air pollution during in utero life. Environmental Health Perspectives 120: 1346–1352. 22626541 10.1289/ehp.1104458 PMC3440109

[61] Janssen BG, Godderis L, Pieters N, Poels K, Kici Ski M, et al (2013) Placental DNA hypomethylation in association with particulate air pollution in early life. Particle and fibre toxicology 10: 22. 23742113 10.1186/1743-8977-10-22 PMC3686623

[62] Shaw CA, Robertson S, Miller MR, Duffin R, Tabor CM, et al (2011) Diesel exhaust particulate–exposed macrophages cause marked endothelial cell activation. American Journal of Respiratory Cell and Molecular Biology 44: 840–851. 20693402 10.1165/rcmb.2010-0011OC

[63] Weldy CS, Wilkerson H-W, Larson TV, Stewart JA, Kavanagh TJ (2011) Diesel particulate exposed macrophages alter endothelial cell expression of eNOS, iNOS, MCP1, and glutathione synthesis genes. Toxicology in vitro : an international journal published in association with BIBRA 25: 2064–2073. 21920430 10.1016/j.tiv.2011.08.008 PMC3217165

[64] Channell MM, Paffett ML, Devlin RB, Madden MC, Campen MJ (2012) Circulating factors induce coronary endothelial cell activation following exposure to inhaled diesel exhaust and nitrogen dioxide in humans: evidence from a novel translational in vitro model. Toxicological Sciences 127: 179–186. 22331494 10.1093/toxsci/kfs084 PMC3327867

[65] Fariss MW, Gilmour MI, Reilly CA, Liedtke W, Ghio AJ (2013) Emerging mechanistic targets in lung injury induced by combustion-generated particles. Toxicological Sciences 132: 253–267. 23322347 10.1093/toxsci/kft001 PMC4447844

[66] Hazari MS, Haykal-Coates N, Winsett DW, Krantz QT, King C, et al (2011) TRPA1 and sympathetic activation contribute to increased risk of triggered cardiac arrhythmias in hypertensive rats exposed to diesel exhaust. Environmental Health Perspectives 119: 951–957. 21377951 10.1289/ehp.1003200 PMC3223009

[67] Dörr J, Fecher-Trost C (2011) TRP channels in female reproductive organs and placenta. Advances in experimental medicine and biology 704: 909–928. 21290333 10.1007/978-94-007-0265-3_47

